# Efficiency analysis of listed pharmaceutical companies in China: A method combining three-stage DEA with undesirable output, PCA, and tobit regression

**DOI:** 10.1371/journal.pone.0329767

**Published:** 2025-08-22

**Authors:** Jiaqiang Sun, Anita Binti Rosli, Adrian Daud

**Affiliations:** 1 Department of Social Science & Management, Faculty of Humanities, Management & Science, Universiti Putra Malaysia Bintulu Campus, Bintulu, Sarawak, Malaysia; 2 Research and Development Center, Fujian Yongjing Technology Co., Ltd., Nanping, Fujian, China; 3 Institute of Ecosystem Science Borneo, Universiti Putra Malaysia, Bintulu, Sarawak, Malaysia; Azad University, IRAN, ISLAMIC REPUBLIC OF

## Abstract

The pharmaceutical industry plays a vital role in safeguarding public health and enhancing industrial competitiveness. However, pharmaceutical industry in China faces persistent challenges, including high pollution, insufficient innovation, and limited profit margins, which constrain its global competitiveness. The current researches on the operational efficiency were either from financial or innovation aspects-lacking a comprehensive assessment framework. Moreover, the impact of environments on efficiency relied on limited indicators and lacked in multicollinearity research. Finally, there was an absence of research on internal resource allocation affects operation efficiency. To fill this, this study aims to evaluate the current operation efficiency of the listed pharmaceutical enterprises in China from finance, innovation, and sustainability, and to reveal the influence environments on efficiency and the impact of the internal resource allocation on efficiency. To achieve these objectives, the study adopts a Three-stage Data Envelopment Analysis with undesirable outputs, integrated with Principal Component Analysis and Tobit regression, to comprehensively evaluate the operational efficiency of Chinese listed pharmaceutical enterprises over the ten-year period from 2013 to 2022 across the aforementioned three dimensions. The findings reveal generally low efficiency with significant regional disparities. North and Northwest China benefit from favorable environmental conditions, while Northeast China suffers from negative impacts. Improvements in external factors, such as innovation, living standards, labor supply, and openness level reduce costs, whereas internal uncontrollable factors, such as state-owned enterprise attributes, increase costs and suppress research and development. In terms of resource allocation, higher management expenses, and personnel allocations decrease efficiency, while increased sales expenditures and improved staff quality enhance efficiency. This study constructs a comprehensive framework for evaluating the operational efficiency and reveals the impact of external environments and internal resource allocation on efficiency. It provides empirical support for policymakers and operation managers seeking to improve the efficiency of the pharmaceutical companies.

## 1 Introduction

The pharmaceutical industry is an integral component of the national economy and public health, playing a crucial role in the healthcare value chain. As the world’s second-largest healthcare market and a region with rapid pharmaceutical sector growth, China’s pharmaceutical industry has achieved notable development over the past two decades. During this period, per capita healthcare expenditure in China increased 11.4 folds, and the overall scale of the pharmaceutical industry expanded 20-fold [1]. However, despite this rapid growth, the Chinese pharmaceutical sector faces numerous pressing issues and challenges that warrant further exploration.

Firstly, the pharmaceutical industry of China is weak in international competitiveness. In the international competitiveness ranking of the pharmaceutical industry, China ranked 47th, which is lower than developing countries such as India and Brazil [2–4]. According to Pharm Exec, in the global pharmaceutical company rankings in 2024, only four Chinese companies entered the top 50 in the world, with the best-ranked Yunnan Baiyao ranking 33rd. This shows that there is a considerable gap between China and the pharmaceutical industry of other countries.

Secondly, insufficient Research and Development (R&D) investment by Chinese pharmaceutical companies has been constrain the development of China’s pharmaceutical industry. Although the R&D-to-revenue ratio of China’s listed pharmaceutical companies has increased from 3.25% in 2013 to 7.21% in 2022, this data is still far lower than that of the United States, Europe and Japan [5]. At the same time, according to data from the National Medical Products Administration (NMPA) official website, in the past ten years, the proportion of innovative drugs in China has been less than 5%, which shows that only pharmaceutical companies in China rely on generic drugs. In the global pharmaceutical industry chain, China and India are both exporters of generic drugs and Active Pharmaceutical Ingredients (APIs) [6,7].

Moreover, the environmental impact of the pharmaceutical industry has become severe [8]. The pharmaceutical production process is complex, involving multi-step chemical reactions, and final product formulation, or extraction by different kinds of organic solvents, all of which generate large volumes of waste, including wastewater, waste gases, and solids [9,10]. These wastes often contain various kinds of chemicals including the final APIs which in low concentration can cause harm to ecosystems [11]. Globally, countries such as the United States, Japan, and European nations are making efforts to address the issue of pharmaceutical pollution [12].

In response to challenges such as insufficient innovation capabilities and serious environmental pollution in the pharmaceutical industry, the Chinese government has issued policies such as *“Green Development of the Pharmaceutical Industry* (2020)” and “*The 14th Five-Year Plan for the Development of the Pharmaceutical Industry* (2022)” [13,14].

Although the pharmaceutical industry in China has insufficient innovation and severe pollution, however, significant gaps exist in the current research for efficiency evaluation. Existing studies predominantly measure efficiency from a single aspect, such as innovation or financial performance, lacking a systematic approach that integrates innovation outcomes, financial indicators, and environmental impacts. These frameworks fail to incorporate undesirable outputs (e.g., wastewater and waste gases) and innovation-specific indicators, thereby neglecting the pharmaceutical industry’s dual characteristics of high pollution and high innovation, leading to inaccuracies in efficiency assessments. Additionally, in the study of the impact of environment on efficiency, although the three-stage DEA method has been widely applied, the environmental indicators applied for analysis often involve only a limited scope, neglecting comprehensive evaluation. And the indicators for the evaluation lack the multicollinearity research, which can affect the accuracy of efficiency evaluation result. Furthermore, for the innovation indicators, the current studies rely on indicators such patent applications or sales from new products, overlooking that Drug Manufacturing Permits also serve as the direct output of innovation capacity in the pharmaceutical industry. Finally, current research on pharmaceutical enterprises lacks empirical analysis of how internal resource allocation specifically affects the operational efficiency. As a result, firms are left without data-driven evidence to support internal management optimization.

To address these research gaps, this study aims to evaluate the operational efficiency of the Chinese pharmaceutical companies comprehensively by integrating financial, sustainability and innovation indicators which including the patent and Drug Manufacture Permit. This study also aims to reveal the impact of external environmental factors and uncontrollable internal environmental factors on operational efficiency, and to analyze the role of internal resource allocation in influencing efficiency, providing corresponding improvement recommendations.

To achieve these objectives, this study adopts a three-stage DEA model with undesirable outputs to comprehensively evaluate the efficiency of Chinese pharmaceutical enterprises by incorporating financial, innovation, and sustainability indicators. Environmental factors are analyzed using Principal Component Analysis (PCA) to extract multidimensional environmental variables. Stochastic Frontier Analysis (SFA) is employed to separate environmental effects, random errors, and managerial efficiency, thereby improving the scientific rigor and accuracy of efficiency evaluations. Furthermore, a Tobit regression model is applied to explore the impact of internal resource allocation on efficiency. Uniquely, this study introduces Drug Manufacturing Permit and Pollution Equivalent as one of the core indicators of innovation and sustainability, which more accurately reflects the innovative and sustainable characteristics of the pharmaceutical industry.

This study presents several key innovations. It constructs a comprehensive efficiency evaluation framework by integrating financial, innovation, and sustainability indicators, which more accurately reflect the dual characteristics of the pharmaceutical industry—namely, high innovation intensity and high environmental pollution. Methodologically, the study combines PCA and SFA to extract multidimensional environmental variables and address multicollinearity, thereby improving the robustness and explanatory power of the regression model while enhancing the accuracy of environmental impact assessment on efficiency. Building on more precise efficiency estimates obtained through the three-stage DEA model with undesirable outputs, the study further applies Tobit regression to examine how internal resource allocation affects firm-level efficiency—an area rarely explored in existing pharmaceutical efficiency research. Additionally, the selection of evaluation indicators is tailored to industry-specific features: Drug Manufacturing Permits and patent authorizations are used to capture both regulatory approval and technological innovation, while Pollution Equivalent is introduced to represent the sector’s complex and intensive pollution profile. Together, these contributions offer a more nuanced and empirically grounded understanding of efficiency in the pharmaceutical industry.

This study is structured into six chapters. Chapter 1 introduces the research background, problem statement and research objectives and operation definition. Chapter 2 presents a literature review, summarizing relevant theoretical frameworks, definitions of efficiency, and common evaluation methods, while comparing their advantages and limitations. Particular attention is given to the strengths of the three-stage DEA method. The chapter also reviews current research on efficiency in the pharmaceutical industry, identifies key gaps, and establishes the conceptual framework for this study. Chapter 3 outlines the research methodology, including data sources, model construction, variable selection, and the application of PCA, SFA, and Tobit models. Chapter 4 reports the empirical results, beginning with multicollinearity diagnostics and Pearson correlation tests to validate the appropriateness of indicator selection. It then presents the first-stage results, principal component extraction and second-stage regression analysis, followed by the estimation of adjusted efficiency scores using the DEA model with undesirable outputs. These refined efficiency scores are then used as dependent variables in Tobit regressions to examine the influence of internal resource allocation. Chapter 5 analyzes the spatial distribution of efficiency, its temporal evolution over the past decade, regional disparities, and the impact of environmental factors across different regions. A comparative analysis with prior studies is also included. Finally, Chapter 6 concludes the thesis by summarizing the main findings, offering policy and managerial implications, acknowledging research limitations, and proposing directions for future research.

In this study, ‘undesirable outputs’ specifically refer to by-products or pollutants generated during the production process. Environmental factors, which include both external environmental elements and uncontrollable internal factors, are collectively referred to as ‘environment’ in this study. The term Drug Manufacturing Permit refers specifically to the administrative license granted by the China Food and Drug Administration (CFDA) that authorizes commercial production. It does not include production approvals issued by the United States Food and Drug Administration (FDA), the European Medicines Agency (EMA), or the World Health Organization (WHO), nor does it include Drug Master File (DMF) registration numbers for Active Pharmaceutical Ingredients (APIs).

## 2 Literature review

### 2.1 Conceptual foundations and definitions of efficiency

Value Maximization Theory posits that industries and enterprises should not only pursue financial performance but also account for the interests of multiple stakeholders, maintaining a dynamic balance between short-term returns and long-term strategic goals. From this perspective, a comprehensive assessment of an industry or firm should encompass both financial indicators and innovation outcomes, thereby capturing the trade-off between immediate performance and sustainable development. Therefore, it is necessary to evaluate the companies from the combination of multiple dimensions [15–17].

Regional Cluster Economic Theory, proposed by Michael Porter, emphasizes that industrial competitiveness is shaped by a combination of factors, including technological capabilities, governmental interventions, and the availability of localized resources. While environmental regulations may initially appear restrictive, they often serve as catalysts for innovation, driving productivity improvements and reinforcing industrial competitiveness. Moreover, the spatial concentration of related firms within regional clusters promotes collaborative networks, facilitates the diffusion of knowledge, and accelerates technological progress. These synergistic effects not only enhance the performance of individual enterprises but also contribute to the broader development of the regional economy. Accordingly, analyzing how regional environmental factors influence industrial efficiency holds considerable theoretical relevance and practical value [18–20].

The Resource-Based View (RBV) is one of the core theories in the field of strategic management, originally proposed by Jay Barney and other scholars in the late 1980s and early 1990 [21]. At its core, the RBV conceptualizes the firm as a collection of unique resources and capabilities that serve as the fundamental source of competitive advantage. Within the RBV framework, the way in which resources are allocated has significant implications for a firm’s efficiency, competitiveness, and long-term development [22–24]. Resource allocation involves not only the distribution of assets but also the integration, optimization, and dynamic adjustment of those resources. RBV provides managers with a theoretical lens through which to analyze competitive advantage from the perspective of internal resources, thereby supporting firms in achieving sustainable development in complex and dynamic market environments [25–29].

Efficiency, as a concept, was first articulated by the Italian economist Pareto, who framed it as an optimal state of resource allocation, commonly called “Pareto optimality”. In this state, no reallocation of resources can increase the benefit of one party without reducing the benefit of others. Pareto optimality fundamentally reveals the economic meaning of efficiency, which is to achieve optimal economic output through the rational distribution of resources. Building on this theory, Farrell (1957) further developed the theory of efficiency by decomposing it into technical efficiency and allocative efficiency. Farrell’s work laid a solid foundation for modern efficiency evaluation theory and provided a theoretical basis for the development of efficiency measurement methods. Efficiency measurement methods are mainly divided into two categories: parametric and non-parametric methods. Among these, Stochastic Frontier Analysis (SFA), as a representative of parametric methods, is commonly used, while Data Envelopment Analysis (DEA) is a typical non-parametric method [30–32].

Efficiency can be defined as an ideal state of resource allocation, where a distribution achieves “Pareto efficiency” (Pareto, 1896), meaning “it is impossible to reallocate resources to make at least one individual better off without making someone else worse off”. This served as the basis for later efficiency research. By distinguishing between technical and allocative efficiency and offering a methodical framework for measurement, Farrell contributed to the advancement of the theory of efficiency evaluation.

### 2.2. The measurement of efficiency: A comparison of SFA, DEA

Currently, efficiency evaluation methods are primarily divided into two kinds: parametric method, such as SFA, which evaluate efficiency by assuming a specific production function [33], and non-parametric approaches, such as DEA, which assess efficiency by constructing a best-practice frontier [34].

The advantages of SFA is the ability to separate inefficiency effects from random noise, which makes it suitable for the situation when the environmental factors or random errors influence efficiency [33,35]. However, One of the disadvantage include the need to specify a functional form for the production function, which can lead to model misspecification if the chosen form does not accurately represent the true production process [36–38].

Non-parametric methods, such as DEA, measure efficiency of the Decision-Making Units (DMUs) in multiple inputs and outputs [34]. Its advantages is that it no need the assumptions of the production function, making it highly flexible for measuring efficiency, especially in in involving complex production relationships [39]. However, its limitations is its inability to account for external environmental factors and random error [35,40].

The three-stage DEA was proposed by Fried et al. (1999) to address the effects of random error and environmental factors on efficiency measurement with multiple inputs and outputs. This method can be divided into three stages, making it possible to effectively distinguish between managerial inefficiencies, random errors, and environmental impacts [40]. The three-stage DEA method overcomes the difficulties caused by random disturbance and environmental influences while using the advantages of non-parametric approaches, such as the capacity to assess several inputs and outputs without the need for a single measurement scale. Additionally, it makes it easier to identify and assess managerial inefficiencies.

Given that both SFA and DEA have their respective strengths and limitations when used independently, scholars have proposed the three-stage DEA model to integrate the advantages of both approaches, thereby enhancing the accuracy and robustness of efficiency evaluation. The three-stage DEA was proposed by Fried et al. (1999) to address the effects of random error and environmental factors on efficiency measurement with multiple inputs and outputs [40]. This method can be divided into three stages, making it possible to effectively distinguish between managerial inefficiencies, random errors, and environmental impacts. The three-stage DEA method overcomes the difficulties caused by random disturbances and environmental influences while leveraging the advantages of non-parametric approaches, such as the capacity to assess multiple inputs and outputs without the need for a single measurement scale. Additionally, it facilitates the identification and assessment of managerial inefficiencies [34,41,42]

Compared to using SFA or DEA alone, the three-stage DEA method offers significant advantages. It retains the key benefit of DEA—namely, the ability to measure efficiency without assuming a specific production function—while its integration with SFA enables the separation of external environmental influences and random noise. As a result, the efficiency scores obtained more accurately reflect the intrinsic efficiency of DMUs [41,43–45]. Owing to these strengths, the three-stage DEA approach has been widely adopted in recent efficiency evaluation research across various sectors [7,46–53].

Furthermore, the integration of the three-stage DEA with Tobit regression further enhances the rigor and practicality of the method. In this approach, the final efficiency derived from the three-stage DEA is used as the dependent variable (DV), while controllable factors, such as external environmental influences and internal resource allocation, are included as independent variables (IV) in the regression analysis. This allows for a more accurate and comprehensive examination of the influencing factors [52,54].

### 2.3 The current research on operational efficiency in the pharmaceutical industry and enterprises

The pharmaceutical industry is a sector with multi-inputs and multi-outputs which includes financial performance, innovation outcomes, and waste production. The advantage of DEA makes this method suitable for efficiency measurement. Moreover, the integration of DEA with methods such as SFA, Tobit regression, and the Malmquist index expand the application of this method in the measurement of efficiency in pharmaceutical companies. For the efficiency measurement of pharmaceutical companies, the current research focuses on two dimensions: innovation and financial performance.

#### 2.3.1 The current research on the innovation efficiency of the pharmaceutical companies.

For innovation efficiency, Xiong & Meng (2019) employed the BCC-DEA model to analyze the innovation efficiency of the listed pharmaceutical companies in China, identifying low pure technical efficiency (PTE) as the main limiting factor [55]. Hao & Ruan (2022), applied the Two-stage DEA, founding the inefficiencies in both the technological development and transformation stages, which attributed to excess Research and Development (R&D) investments and patent applications [56]. Similarly, Qiu et al. (2023) adopted a Three-stage DEA to evaluate the innovation efficiency of China’s pharmaceutical industry, revealing low overall innovation efficiency with uneven distribution [57].

International research about pharmaceutical innovation efficiency has also gained traction. For instance, Shin et al. (2018) employed SFA and multi-frontier analysis to assess the efficiency of 705 U.S. pharmaceutical companies [58]. They concluded that different open innovation strategies had varying impacts on firms, with “inside-out” strategies performing best in terms of innovation and efficiency. Schuhmacher et al. (2021) used new molecular entities (NMEs) as output indicators and identified Pfizer and Merck as the most efficient firms globally [59]. However, Schuhmacher et al. (2023) later pointed out that these global giants now face significant challenges in R&D efficiency [60].

There are notable differences in output indicators between Chinese and international studies. Chinese research predominantly utilizes patent applications and new product sales as output metrics, whereas international studies adopt a broader range of indicators, including NME approvals, publication impact factors, and financial metrics. According to the *Drug Registration Regulation* (2020 edition) in China, drugs must obtain manufacturing licenses to be marketed, which has made R&D investments primarily oriented toward achieving this goal. Consequently, metrics such as patent applications or academic publications fail to reflect innovation outputs fully. Although NMEs can partially represent innovation outcomes, their proportion in China remains below 5%, limiting their reliability as innovation indicators.

#### 2.3.2 The Current Research on the Financial Efficiency of the Pharmaceutical Companies.

Financial performance is another critical dimension for evaluating operational efficiency in pharmaceutical enterprises. Cai and Sun (2013), through the combined use of DEA and SFA, demonstrated that increases in technical knowledge reserves significantly enhance corporate revenues [4]. Similarly, Chen et al. (2015), combining slack-based measurement (SBM) and an adjusted residual income model, reached comparable conclusions [61]. Xia et al. (2022) employed the BCC-DEA model to analyze the operational efficiency of Chinese-listed pharmaceutical firms, observing a downward trend in overall financial efficiency [62]. Only the bio-pharmaceutical sector showed an upward trend, while the efficiency of the chemical pharmaceutical and TCM sectors declined. Additionally, Lin et al. (2021) utilized a two-stage network DEA and the Malmquist index, finding no significant impact of government subsidies on financial efficiency [63]. Yang (2024), through a three-stage DEA and Malmquist index, revealed that the overall efficiency of Chinese pharmaceutical enterprises is low and exhibits significant interannual fluctuations [64].

In comparison, international studies employ a wider range of indicators. Gascón et al. (2017) combined financial and innovation metrics to study the efficiency of large U.S. pharmaceutical firms, highlighting the intense competition in the industry and a small efficiency gap between high- and low-performing decision-making units (DMUs) [65]. Hamad and Tarnoczi (2021) used the Value Added Intellectual Capital model to evaluate pharmaceutical efficiency in the Visegrad countries (Czech Republic, Hungary, Poland, and Slovakia). Slovakia demonstrated superior performance in human capital efficiency, structural capital efficiency, and overall intellectual capital efficiency. Similarly, Riaz et al. (2023) applied DEA combined with financial output indicators, such as revenue, earnings per share, dividends per share, and return on equity, to evaluate the efficiency of pharmaceutical firms in Pakistan [66].

### 2.4 The research gap in the efficiency evaluation of pharmaceutical companies

Despite existing studies on the efficiency of pharmaceutical enterprises, there remains a significant research gap.

#### 2.4.1 The lack of comprehensive evaluation frameworks and overlooking the characteristics in the evaluation the operation efficiency.

Pharmaceutical enterprises are characterized by both high levels of innovation and significant environmental pollution. However, current research on the operational efficiency of pharmaceutical companies in China focuses on a single dimension—either financial performance or innovation—without integrating these characteristics into a comprehensive evaluation framework. As a result, the dual nature of the pharmaceutical industry, namely “high pollution and high innovation,” is often overlooked, leading to potential biases in efficiency assessments. To date, only Gascón et al. (2017) have incorporated both financial and innovation dimensions in evaluating pharmaceutical firms’ operational efficiency; no other studies have conducted a comprehensive multidimensional analysis [65]. This contrasts sharply with efficiency research in other pollution-intensive industries, where multidimensional frameworks are more commonly applied [67,68]

It is important to note that financial indicators primarily capture a firm’s short-term profitability, innovation reflects its long-term growth potential, and sustainability represents both corporate social responsibility and a foundation for stable operations. Evaluating these dimensions in isolation fails to fully capture the overall operational performance of pharmaceutical enterprises.

Moreover, the innovation indicators, existing studies commonly use metrics such as new product sales, the number of patent applications, new molecular entities (NMEs), and journal impact factors to assess the innovation efficiency of pharmaceutical firms. However, according to China’s Drug Administration Law and Drug Registration Regulation of China, these indicators do not fully reflect the regulatory-driven objectives of pharmaceutical innovation. In fact, the acquisition of a Drug Manufacturing Permit is a legal prerequisite for bringing a drug to market and should be considered a core output of innovation performance in the Chinese context. Nonetheless, this critical indicator has been largely overlooked in current research, resulting in a disconnect between academic evaluation metrics and the actual innovation goals shaped by regulatory requirements.

#### 2.4.2 Current research on impact of environmental factors to efficiency was limited.

The current research on the impact of environmental factors on firm efficiency remains limited in scope and depth. Although several studies have employed the three-stage DEA method to investigate the influence of environmental conditions, there is a prevalent tendency to use single proxy variables to represent complex environmental dimensions, which constrains the comprehensiveness of the analysis. For instance, Qiu et al. (2023) used the number of employees to represent firm size, the number of employees with a bachelor’s degree or higher to indicate employee quality, and Return on Equity (ROE) and the leverage ratio (LEV) as environmental indicators [57]. Similarly, Yang (2024) employed government subsidies, per capita GDP, and firm age to capture environmental effects on efficiency [64]. Sun et al. (2024) constructed environmental indicators using per capita disposable income to reflect wealth levels, the working-age population to indicate labor supply, and local GDP to represent regional economic development [7].

While these studies offer valuable perspectives on the relationship between environmental factors and efficiency, they remain insufficient in capturing the full complexity of environmental influences. Environmental conditions are inherently multidimensional and dynamic; relying on single indicators fails to reflect their comprehensive impact. Moreover, including an excessive number of environmental variables in regression models can lead to multicollinearity issues, thereby compromising the accuracy and reliability of the estimation results [69–71].

#### 2.4.3 Insufficient studies combination the environmental factors and resource allocation.

The Resource-Based View (RBV) posits that the uniqueness of a firm’s resources and the effectiveness of their allocation are critical determinants of sustained competitive advantage. However, the impact of internal resource allocation on operational efficiency remains underexplored in the existing literature, with limited empirical investigation in this area. Moreover, current studies on efficiency determinants tend to examine the external environment. While external environmental factors are largely uncontrollable, internal resource allocation represents a managerial lever that can be strategically adjusted. A comprehensive analysis that integrates both external and internal dimensions would offer deeper insights into the mechanisms driving efficiency and provide a more robust foundation for evidence-based efficiency improvement strategies.

In conclusion, the research gaps highlight the necessity of developing a framework that combines financial, innovation, and sustainability dimensions while incorporating adaptive indicators tailored to the unique characteristics of Chinese pharmaceutical enterprises. Such research not only addresses the limitations of current methodologies but also provides valuable insights for enhancing the operational efficiency and sustainable development of the pharmaceutical industry.

### 2.5 The conceptual framework of this study

**Fig 1:**
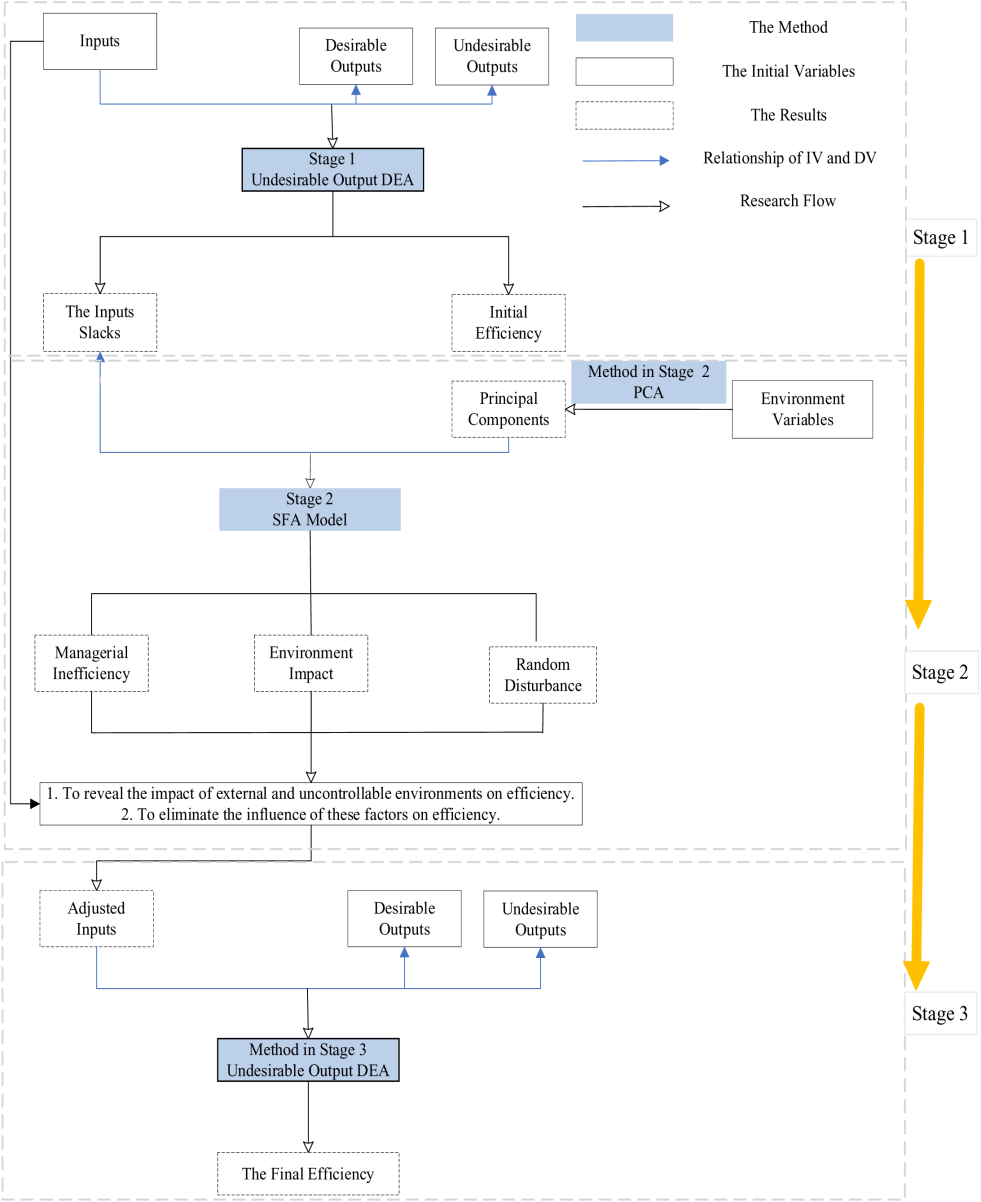
Conceptual framework of this study.

Based on the research gap, the study has three objectives. The first one is to measure the operational efficiency of the listed pharmaceutical companies in China from financial, innovation and sustainable development dimensions with more proper indicators. The second objective is to reveal the impact of comprehensive environmental factors on operational efficiency. And the third is to reveal the how the internal resource allocation impact the operation efficiency. To achieve these research objectives, this study employs a three-stage DEA model combined with PCA and Tobit regression to evaluate the operational efficiency of listed pharmaceutical companies. Fig 1 presents the conceptual framework of this study, adapted from Zhao et al. (2019), with modifications to include undesirable outputs and PCA [72].

The procedure can be divided into 4 stages.

Stage 1: Initial Efficiency and Slacks of inputs Measurement. In this stage, DEA with undesirable output is used to measure the efficiency values of the listed pharmaceuticals companies. The main objective of this stage is to obtain the current efficiency distribution and calculate the slacks of input variables.

Stage 2: To reveal the Environmental Factor impacts the Efficiency. This stage consists of two steps. In the first step, PCA is employed to extract principal components from multiple potential environmental variables and to reduce multicollinearity.

In the second steps, the extracted environmental variables are used as independent variables (IV), and the slacks of the inputs from Stage 1 are used as the dependent variable (DV) in the SFA regression, revealing how the environment influences the slacks and efficiency.

Stage 3: Recalculation and Obtaining the Accurate Efficiency. In this stage, the adjusted inputs are obtained in Stage 2, and the original output, will be employed to recalculate and obtain accurate operation efficiency using undesirable output DEA.

Stage 4: Revealing the Influence of the Internal Resource Allocation on Efficiency. In this stage, the accurate efficiency which was obtained from the previous stage are used as the DV, while the internal resource allocation ways of the firm are used as the IV in the regression analysis, thereby revealing the impact of resource allocation on efficiency. Given that efficiency data exhibit censoring or truncation characteristics, the Tobit regression model is employed in this study. The analysis in this stage provides management implication based on empirical evidence for operational managers.

The conceptual framework of this study is primarily based on the theoretical frameworks of Zhao et al. (2019) and Zhang & Cui (2024), which form the analytical model for this research [52,72], as well as the PCA-DEA approach proposed by Sun et al, [7]. Fig 1 illustrates the conceptual framework of this study.

## 3 Methods and materials

### 3.1 The undesirable output DEA Mode

#### Stage 1: Undesirable output DEA.

In the pharmaceutical industry, materials mainly consist of chemical substances, such as key starting materials, excipients, solvents, and catalysts. Through a series of complex chemical reactions, these materials are gradually transformed into target products, such as APIs. However, due to the incomplete nature of chemical reactions and the complexity of post-reaction processing, multiple undesirable outputs are generated along with the target product. These undesirable outputs include not only by-products but also polluted solvents, wastewater containing chemical substances, solid waste, and volatile organic compounds (VOCs). These outputs are unavoidable in pharmaceutical processes, posing significant challenges for both environmental protection and operational management [73–76].

In the presence of undesirable outputs, several approaches have been proposed for their treatment, including being disregarded, converted into input variables (e.g., S-Z transformation), subjected to hyperbolic transformation or addressed through the directional distance function method. However, these methods are characterized by certain limitations, such as insufficient theoretical support and computational complexity [77,78].

To evaluate the efficiency of the pharmaceutical industry, this study employs a DEA model with undesirable outputs, which integrates multiple inputs, desirable outputs, and undesirable outputs within a unified framework to assess overall efficiency [79].

In a set of DMUs with both desirable and undesirable outputs, the output Y can be decomposed into desirable outputs (${\text{y}}^{\text{g}}$) and undesirable outputs (${\text{Y}}^{\text{b}}$), with input variables represented as X. Both X and Y are defined as non-zero and non-negative. The equation (1) illustrate the production possibility of this DMUs.


$$\text{P = }\left\{\left(\text{x,}{\text{y}}^{\text{g}}\text{,}{\text{y}}^{\text{b}}\right)|x\geq X\lambda ,{\text{y}}^{\text{g}}\leq {\text{Y}}^{\text{g}}\lambda ,{\text{y}}^{\text{b}}\geq {\text{Y}}^{\text{b}}\lambda ,L\leq e\lambda \leq U,\lambda \geq 0\right\}$$ (1)

Where  λ is the intensity vector, and L and U are the lower and upper bounds of the intensity vector, respectively.

In a DMU with both desirable and undesirable outputs, the total output weight is defined as 1, where the total weight for desirable outputs is ${\;W}_{\text{1}}$ and the total weight for undesirable outputs is ${\text{W}}_{\text{2}}$. Thus, in the DEA model, the W1 > 0, W2 > 0, W1 + W2 = 1. The efficiency of the DMU can be defined as follow:


$$\rho^{*}\text{=min}\frac{\text{1 - }\frac{\text{1}}{\text{m}}\sum_{\text{i = 1}}^{\text{m}}\frac{{\text{s}}_{\text{io}}^{\text{ - }}}{{\text{x}}_{\text{io}}}}{\text{1 + }\frac{\text{1}}{\text{s}}\left({\text{W}}_{\text{1}}\sum_{\text{r = 1}}^{{\text{s}}_{\text{1}}}\frac{{\text{s}}_{\text{r}}^{\text{g}}}{{\text{y}}_{\text{ro}}^{\text{g}}}\text{ + }{\text{W}}_{\text{2}}\sum_{\text{r = 1}}^{{\text{s}}_{\text{2}}}\frac{{\text{s}}_{\text{r}}^{\text{b}}}{{\text{y}}_{\text{ro}}^{\text{b}}}\right)}\;Subject\;to:\;\begin{array}{c} \sum_{j=1}^{n}\lambda_{j}x_{ij}+s_{i}^{-}=x_{i},i=1,\ldots ,m\\ \sum_{j=1}^{n}\lambda_{j}y_{grj}-s_{g}^{+}=y_{gr},r=1,\ldots ,g\\ \sum_{j=1}^{n}\lambda_{j}y_{bzj}+s_{b}^{-}=y_{bz},z=1,\ldots ,b\\ \lambda_{j}\geq 0,s_{i}^{-},s_{g}^{+},s_{b}^{-}\geq 0,\forall j \end{array}$$ (2)

In this study, there are 6 output indicators in total, which include five desirable outputs from the financial, and innovation dimensions, and one eco-environment pollution indicator for the undesirable outputs. To balance the importance of the good and bad outputs, the total weight for the desirable outputs is set to 0.8, reflecting their importance to the companies, while the weight for the undesirable outputs is set to 0.2, emphasizing their impact on efficiency while avoiding an excessive influence on the overall evaluation. This weight set ensures a holistic measurement according to the objectives of this study [7].

### 3.2 Stage 2: The SFA model

According to Michael Porter’s theory of industrial clusters, enterprises in specific regions can promote industrial synergy and development through shared resources. China has already formed several industrial clusters, such as the Yangtze River Delta Economic Zone and the Pearl River Delta Economic Zone. However, as the Chinese government promotes environmental protection, the environmental capacity of various regions is getting smaller. Especially in developed areas, such as Shanghai, Shenzhen, Beijing, Suzhou and other areas, these industrial clusters cannot continue to engage in certain industries indefinitely, especially high-pollution industries like the pharmaceutical industry. Developed regions continue to improve standards and give priority to the development of environmentally friendly industries, which poses greater challenges to the operations of local pharmaceutical companies.

To reveal the impact of the environment on efficiency while eliminating its influence, this stage employs SFA to decompose the input slacks into management inefficiency, environmental effects, and random disturbances.

Since numerous factors may influence efficiency and there could be severe multicollinearity among these variables, dimensionality reduction is performed before applying SFA. The main components are extracted, and environmental factors (Z) are determined based on their variance contribution rates. These environmental factors will be utilized in the subsequent regression analysis to reveal their impact on inefficiency.

The next step in Stage 2 is to perform SFA regression, using the environmental factors (Z) as independent variables and the input slacks obtained in the first stage as dependent variables.The regression is represented by Equation (3):


$${\text{S}}_{\text{ij}}\text{ = f}\left({\text{Z}}_{\text{j}}\text{,}\beta_{\text{i}}\right)\text{ + }{\text{v}}_{\text{ij}}\text{ + }\mu_{\text{ij}},j=1,2,\cdots ,M;i=1,2,\cdots ,N\mathrm{\backslash ]}$$ (3)

Among them, ${\text{S}}_{\text{ij}}$ represents i-th input salcks for the j-th DMU. ${\;Z}_{\text{j}}$ represents the environmental factors in j-th DMU, and $\beta_{\text{i}}$ represents their corresponding coefficients. Thus, $\text{f}\left({\text{Z}}_{\text{j}}\text{,}\beta_{\text{i}}\right)$ indicating the impact of environmental factors on input slack, which are generally assumed that $\text{f}\left({\text{Z}}_{\text{j}}\text{,}\beta_{\text{i}}\right)\text{ = }{\text{Z}}_{\text{j}}\beta_{\text{i}}$, and the ${\text{v}}_{\text{ij}}\text{ + }\mu_{\text{ij}}$ represents the composite error term ($\epsilon_{\text{ij}}$), which includes the random error term (${\text{v}}_{\text{ij}}$) and management inefficiency term ($\mu_{\text{ij}}$). The random error ${\text{v}}_{\text{ij}}$ follows a symmetric normal distribution $\text{N}\left(\text{0,}\sigma_{\text{v}}^{\text{2}}\right)$, representing statistical noise, while the $\mu_{\text{ij}}$ follow a non-negative truncated normal distribution $\text{\textbackslash{} }{\text{N}}^{\text{ + }}\left(\text{0,}\sigma_{\text{u}}^{\text{2}}\right)$ accounting for inefficiency effects.

Each of the two following a specific distribution, and they are independent of each other. This allows us to derive the proportion of technical inefficiency variance to the total variance. Additionally, the parameters and error terms can be estimated using the Maximum Likelihood Estimation (MLE) method or the Adjusted Least Squares (ALS) method, thereby obtaining ${\text{v}}_{\text{ij}}$ and $\text{\textbackslash{} }\mu_{\text{ij}}$. Based on the distribution assumptions of environmental factors and random disturbances, technical inefficiency can be calculated using Equation (4).


$$\text{E}(\mu |\epsilon )=\sigma^{*}*[\frac{\varphi (\lambda \frac{\epsilon }{\sigma })}{\Phi (\frac{\lambda \epsilon }{\sigma })}+\frac{\lambda \epsilon }{\sigma }]$$ (4)

in the Equation (4), where:


$$\epsilon ={\text{S}}_{\text{ij}}-\text{ f}\left({\text{Z}}_{\text{j}}\text{,}\beta_{\text{i}}\right)$$

$$\sigma_{\mu }\text{ = }\sqrt{\left(\gamma *\sigma^{\text{2}}\right)}$$

$$\sigma_{\text{v}}\text{ = }\sqrt{\sigma^{\text{2}}-\sigma_{\mu }^{\text{2}}}$$

$${\;\sigma }^{*}\text{ = }{(\sigma }_{\mu }*\sigma_{\text{v}})/\sigma$$

$${\lambda =\sigma }_{\mu }\text{/}\sigma_{\text{v}}$$

The random error term can be calculated using the Equation (5).


$${\text{v}}_{\text{ij}}\text{ = }{\text{S}}_{\text{ij}}\text{ - }{\text{Z}}_{\text{j}}\hat{\beta_{\text{i}}}\text{ - }\hat{\text{E}}\left({\text{v}}_{\text{ij}}|{\text{v}}_{\text{ni}}\text{ + }\mu_{\text{ij}}\right),j=1,2,\cdots ,M;i=1,2,\cdots ,N\mathrm{\backslash ]}$$ (5)

Finally, the readjusted inputs can be got as from Equation (6):


$$\hat{{\text{x}}_{\text{ij}}}\text{ = }{\text{x}}_{\text{ij}}\text{ + }\left[{\text{max}}_{\text{n}}\left\{{\text{Z}}_{\text{j}}\hat{\beta_{\text{i}}}\right\}\text{ - }{\text{Z}}_{\text{j}}\hat{\beta_{\text{i}}}\right]\text{ + }\left[{\text{max}}_{\text{n}}\left\{\hat{{\text{v}}_{\text{ij}}}\right\}\text{ - }{\text{v}}_{\text{ij}}\right]$$ (6)

In Equation (6), $\hat{{\text{x}}_{\text{ij}}}$ represents the adjusted input factors, $\hat{\beta_{\text{i}}}$ represents the estimated coefficients of the external environmental variables, and ${\text{v}}_{\text{ij}}$ represents the estimated values of the random disturbance term.

### 3.3 The design of the variables

#### 3.3.1 Input indicators.

This study constructs the input variables from three different dimensions, that are total asset, operation cost and the number of employees [63–65].

**Total Assets (X1):** This variable indicates the scale and capital structure of a firm, representing the total financial resources available for production activities. It serves as a fundamental input to evaluate a company’s operational foundation and investment intensity.

**Operating Costs (X2):** Operating costs include expenditures incurred during production, such as raw material procurement, energy usage, and manufacturing management. These costs reflect the magnitude of capital consumption and the operational efficiency of the enterprise.

**Number of Employees (X3):** This variable captures the human capital engaged in production and management. It reflects the size of the labor force and the extent of manpower deployment within the organization.

**R&D Investment (X4):** R&D investment denotes the firm’s funding allocated to research and development. It illustrates the strategic focus on innovation, long-term competitiveness, and the pursuit of technological advancement.

#### 3.3.2 Output indicator.

This study constructs a total of 5 output indicators across three dimensions: financial, innovation, and sustainable development. Among these, three are financial indicators, two are sustainable development indicators, and one is an innovation indicator. The details are as follows:

Y_1_. Operating Revenue: In this study, operating revenue refers to the income that is from the main business. It represents the DUMs’ market capability [65].

Y_2_. Earnings Before Interest and Taxes (EBIT): Due to different tax policies in variate districts and the debt ratio variation in the DMUs, the EBIT, compared to the profit is a more suitable indicator of the profitability [80,81].

Y_3_. Net Operating Cash Flow (NOCF): This indicator is the core metric for the ability to deal wiht the financial risks and ensures operation of business activities [63].

Y_4_. Number of Patent Approval: This indicator is one of the metrics for those intermediaries or APIs that sell to the non-regulated market, and the approved patent is one of the new technology innovations [63].

Y_5_. Drug Manufacturing Permit: This indicator is a direct output of R&D investment for pharmaceutical companies as launch into the market as requirement by the NMPA [59,65].

Y_6_. Wastewater Pollution Equivalent: This study employs Wastewater Pollution Equivalent as an new indicator for undesirable output. Due to the difference in the composition of pollutants across regions and enterprises, and the differences in local wastewater acceptance standards and emission regulations, these indicators are obtained based on China’s relevant legal standards and the equation for those indicators are presented as Equation 7 [7,82].


$$\;Pollution\;Equivalent=\sum_{\text{i = 1}}^{\text{n}}\left({\text{C}}_{\text{i}}\text{/}{\text{F}}_{\text{i}}\right)\mathrm{\backslash ]}$$ (7)

$C_{i}$ (Pollutant Emission), represents the amount or concentration of i - th pollutants emitted by a DMU, typically expressed in appropriate units such as tons, or kilograms.

$F_{i}$ (Equivalent Factor), represents the Equivalent Factor of the i-th pollutant, which measures its relative harmfulness compared to a reference pollutant. $F_{i}$ is a non-negative, non-zero number; the smaller the value of $F_{i}$, the greater the pollution.

In the variable selection process, Pearson correlation analysis will be employed to examine the relationships between input variables and desirable output variables. The results indicate that input variables are significantly correlated with desirable outputs (p < 0.05), which aligns with the fundamental assumption of positive relationships in DEA modeling [83]. In contrast, no significant correlation was observed between input variables and undesirable outputs (p > 0.1), further supporting the validity of the variable classification. The detailed analysis and empirical results are presented in Section 4.2 of this study.

#### 3.3.3 Environmental variables.

Based on Michael Porter’s theory of regional clustering, the theory of comparative advantage, and regional economic theory, external factors such as local economic, cultural, and technological environments, as well as internal factors within the firm, may influence the operational environment of the enterprise [84–87]. Therefore, in the first step of SFA, Principal Component Analysis (PCA) is employed to extract environmental variables. The principle for selecting the original environmental factors is to identify indicators that may influence the daily operations of a firm but cannot be controlled by the firm itself. Therefore, this study primarily considers macro factors such as economic, cultural, and local technological innovation factors, as well as certain internal factors that cannot be improved through managerial actions. In the economic environment, indicators such as the local economic level, income level, fiscal revenue, and investment levels directly affect the operational costs of pharmaceutical companies. In terms of social factors, population structure and educational levels also directly influence the workforce and automation control levels in the pharmaceutical industry, which may, in turn, affect the company’s costs and innovation capabilities. Local investments in technology, environmental protection, and education can also directly impact the innovation levels of local enterprises and their capacity for pollution control. Additionally, certain internal factors, such as the company’s ownership structure, nature, and size, may affect decision-making, costs, and innovation within the firm [7,57,88–90].

Based on this principle, this study selects 20 external environmental factors from the economic, cultural, and technological dimensions as potential environmental indicators, along with three internal factors as key influences on the operational performance of pharmaceutical companies. Principal Component Analysis (PCA) was used to extract five principal components, which are: Innovation and Innovation Environment (Z1), Living Standards and Environment (Z2), Labor Suitable for The Pharmaceutical Industry (Z3), Foreign Investment Level (Z4), Internal Corporate Environment (Z5), all of which are listed in Table 1.

**Table 1:** Input, output, and environment indicators in the study.

Type	Measurement Dimension	Indicators Name	Unit
Input Indicators	Capital	X_1,_ Total assets	Million CNY
	Operation cost	X_2,_ Main business costs	Million CNY
	labour	X_3,_ Number of employees	Person
	Emphasize on innovation	X_4_ R&D investment	Million CNY
Output Indicators	Market Performance	Y_1,_ Operating Revenue	Million CNY
	Profitability	Y_2_, EBIT	Million CNY
	Risk control capability	Y_3_, NOCF	Million CNY
	Innovation in technology	Y_4_, Patent approval	Units
	Innovation in drug manufacturing	Y_5_, Drug Manufacturing Permit	Units
	Sustainability	$Y_{6}^{b}$, Waste Pollution Equivalent	t
Environment Indicators^1^	Z1: Innovation and Innovation Environment		–
	Z2: Living Standards and Environment		–
	Z3: Labor Suitable for The Pharmaceutical Industry		–
	Z4: Foreign Investment Level		–
	Z5: Internal Corporate Environment		–

^1^ The environmental indicators are derived from the results of the PCA analysis in Section 4.3.1.

The Table 1 presents the input indicators, output indicators, and environmental indicators selected or derived in this study.

### 3.4 Data collection

Sample Scope: The sample includes pharmaceutical companies listed on the Shenzhen Stock Exchange (SZSE) and the Shanghai Stock Exchange (SSE), with the C27 category in the ShenWan Industry Classification, and all the companies are Chinese companies. Due to the unavailability of data from Taiwan, Hong Kong, and Macau through public channels, and the absence of listed pharmaceutical companies in Qinghai and Ningxia, this study excludes these five regions. In this study, totally 1745 DMUs are included.

Time of the Data: All the data was from 2013 to 2022, except the Drug Manufacturing Permit and Patent form 2014–2023, as drug approvals have a one-year lag period (Data from NMPA), as well as the patent approval [91].

Sample Selection: To ensure consistency and reliability, DMUs with long-term losses or under special treatment (ST) are excluded as they operate under atypical conditions that could distort efficiency results.

Data Processing: All data were processed to ensure non-negativity, and the data for PCA are normalized using the Min-Max normalization method before being input into the PCA analysis.Financial data of DMUs was from the annual reports and social responsibility reports of listed companies, and is available at https://www.wind.com.cn. The numbers of Drug Manufacturing Permit are accessible from https://www.nmpa.gov.cn. Environmental variables are accessible at https://www.stats.gov.cn. Table 2 presents the descriptive statistics of the data sample used in this study.

**Table 2:** The descriptive statistics of the data sample used in this study.

Variable	Maximum	Minimum	Mean	Standard deviation
X_1,_ Total Assets (Million CNY)	107163.91	286.71	6466.61	8740.17
X_2,_ Main Business costs (Million CNY)	57510.95	5.64	2187.51	4182.76
X_3,_ Number of Employees (Person)	38399	68	3557	4316
R&D investment (Million CNY)	6346	0.93	194.6	417.0
Y_1,_ Operating Revenue (Million CNY)	70788.16	120.68	3516.39	5700.81
Y_2_, EBIT (Million CNY)	14417.00	0.00	3011.87	952.77
Y_3_, NOCF (Million CNY	9627.59	0.00	1593.05	785.01
Y_4_, Patent Approval (Unites)	479	0	23.63	34.70
Y_5_, Drug Manufacturing Permit (Unites)	196	0	8.17	17.10
$Y_{6}^{b}$ Wate Pollution Equivalent (t)	1105.31	6.81	468.75	279.08

Data Processing: All data used for the DEA analysis has undergone non-negativity and zero-adjustment processing to ensure the accuracy of the model calculations.

The software used in this study: DEA-Solver 13C, Frontier 4.1, and SPSS 27.0.1.0. All of the software is free version.

## 4. Result

### 4.1 Multicollinearity assessment for inputs and outputs

In DEA, multicollinearity may result in significant fluctuations and instability in the results [92]. Therefore, this study employed the variance inflation factor (VIF) to separately examine the input and output variables for multicollinearity, ensuring the absence of severe multicollinearity among the variables and thereby guaranteeing the robustness and reliability of the results. Table 3 presents the VIF results for the input variables.

**Table 3:** Results of multicollinearity assessment for inputs.

Variable	Total asset	Operation cost	Employee number	R&D Investment
Total asset	–	3.407^***^	5.036^***^	3.794^***^
Operation cost	2.114^***^	–	2.983^***^	2.566^***^
Employee number	3.233^***^	3.085^***^	–	3.105^***^
R&D investment	1.815^***^	1.979^***^	2.315^***^	–

Note: ^***^ and ^**^ represent 99% and 95% confidence levels, respectively

The VIF value of 5.036 for total asset and employee number reaches the cautionary threshold, suggesting a moderate level of correlation between these two variables. However, as the value does not exceed the threshold of 10, it does not indicate severe multicollinearity. Given their common usage as key input indicators in economic and operational analysis, total asset and employee number are retained in the model.

Similarly, this study conducted VIF tests on the output variables to ensure the absence of severe multicollinearity among them. Table 4 presents the results of the multicollinearity tests for the output variables.

**Table 4:** Results of multicollinearity assessment for outputs.

Variable	Operation revenue	EBIT	NOCF	Approved patent	Waste Equivalent	Drug Manufacturing Permit
Operation revenue	–	2.274^***^	2.138^***^	2.549^***^	2.481^***^	2.395^***^
EBIT	2.522^***^	–	2.022^***^	3.335	2.746^***^	2.772
NOCF	2.51^***^	2.14^***^	–	3.285^***^	2.924^**^	2.932
Approved patent	1.333^***^	1.328^***^	1.332^***^	–	1.339	1.228^***^
Waste equivalence	1.015^***^	1.013^***^	1.019^**^	1.0172	–	1.015^***^
Drug Manufacturing Permit	1.237^***^	1.291	1.290	1.117^***^	1.281^***^	–

Note: ^***^ and ^**^ represent 99% and 95% confidence levels, respectively.

The Table 4 shows that the VIF values for the outputs, including operation revenue, EBIT, NOCF, Drug Manufacturing Permit and Water Pollution Equivalent are below 5, indicating a low degree of multicollinearity among these variables.

In summary, by performing VIF tests on input and output variables, this study ensures the absence of severe multicollinearity among the variables, thereby enhancing the scientific validity of the DEA model, the accuracy of efficiency measurements, and the reliability of the analytical conclusions.

### 4.2 Correlation verification of input-output variables

In DEA, it is crucial to ensure directional consistency between input and output variables. Specifically, for desirable outputs, an increase in input variables should generally lead to an increase in output variables, reflecting a positive correlation. Conversely, for undesirable outputs, inputs should not exhibit a significant positive correlation with outputs, as this would contradict the theoretical assumptions of the model [83].

In this study, Pearson correlation analysis is employed to examine the relationships between inputs and outputs. This step is conducted to verify that the selected indicators meet the requirement of directional consistency, thereby ensuring the validity and appropriateness of the DEA model. The Table 5 presents the results of the Pearson correlation analysis.

**Table 5:** Pearson correlation test results for input-output variables.

Variables	Y1 Operation Revenue	Y2 EBIT	Y3 NOCF	Y4 Approved patent	Y5 Drug Manufacture Permit	Y6 (bad) Waste Equivalent
X1 Total Asset	.876^***^	.751^***^	.705^***^	.391^***^	.425^***^	0.013
X2 Operation Cost	.934^***^	.592^***^	.569^***^	.441^***^	.342^***^	−.051^*^
X3 Employee Number	.789^***^	.604^***^	.605^***^	.492^***^	.532^***^	−.048^*^
X4 R&D Investment	.560^***^	.583^***^	.542^***^	.679^***^	.379^***^	.062^*^

Note: ^***^, ^**^ and ^*^ represent significance levels of 99%, 95%, and 10% confidence, respectively.

The four input variables exhibit statistically significant positive correlations (p < 0.01) with the five desirable output variables, satisfying the fundamental requirement of the DEA method regarding directional consistency. This indicates that the selected input variables have strong explanatory power for the outputs and that the input-output relationship is appropriately specified. In contrast, the correlation coefficients between the input variables and the pollution equivalent (undesirable output) are all below 0.1 and statistically insignificant (p > 0.05), as indicated by the t-tests. This suggests that there is no direct relationship between input levels and pollution generation. These results confirm the appropriateness of the input and output variable selection and further justify the use of an undesirable-output DEA model in this study.

### 4.3 The result of stage 1: The current operation performance of pharmaceutical companies

After processing the data to ensure non-zero and non-negative values, the Undesirable Output DEA model in DEA-Solver (13C) was applied to measure the PE, PTE, SE. Table 6 presents the test results from the first and third stages.

**Table 6:** The result of the stage 1 and stage 3 in undesirable output DEA.

Region		The efficiency in Stage1			The efficiency in Stage3		
		TE	PTE	SE	TE	PTE	SE
National wide	Mean	0.202	0.287	0.732	0.227	0.260	0.891
	SD	0.279	0.332	0.244	0.297	0.323	0.163
	Effective DMUs	144	235	144	167	208	167
North China (Beijing, Tianjin, Hebei, Shanxi, Inner Mongolia)	Mean	0.264	0.337	0.791	0.217	0.238	0.924
	Effective DMUs	29	43	29	15	19	15
Northeast China (Liaoning, Jiling, Heilongjiang)	Mean	0.192	0.309	0.697	0.235	0.280	0.907
	Effective DMUs	6	13	6	5	10	5
East China (Shanghai, Jiangsu, Zhejiang, Anhui, Fujian, Jiangxi, Shandong)	Mean	0.188	0.275	0.731	0.231	0.263	0.892
	Effective DMUs	53	86	53	76	92	76
Central China (Hunan, Hubei, Henan)	Mean	0.234	0.347	0.715	0.286	0.318	0.897
	Effective DMUs	14	25	14	19	22	19
South China (Guangdong, Guangxi, Hainan)	Mean	0.197	0.295	0.682	0.233	0.286	0.848
	Effective DMUs	17	26	17	21	27	21
Southwest China (Chongqing, Sichuan, Guizhou, Yunnan, Tibet)	Mean	0.197	0.295	0.682	0.233	0.286	0.848
	Effective DMUs	20	37	20	25	31	25
Northwest China (Shaanxi, Qinghai, Ningxia, Gansu, Xinjiang)	Mean	0.212	0.216	0.768	0.127	0.165	0.810
	Effective DMUs	5	5	5	6	7	5

Note: The average value is obtained by calculating the efficiency of each companies using DEA and then averaging the efficiency values by region

In the first stage of the DEA analysis, the average efficiency of Chinese pharmaceutical enterprises was 0.202, and only 8.25% of the DMUs were found to be DEA-efficient over the ten years. Geographically, the highest efficiency was observed in the North China region, at 0.264, followed by the Central China region at 0.234. In contrast, the lowest efficiency was found in the East China and Northeast China regions, with values of 0.188 and 0.192, respectively. This indicates that, overall, the efficiency of Chinese pharmaceutical enterprises is relatively low.

Further analysis reveals that the standard deviation is 0.279, while the average efficiency is 0.202, resulting in a coefficient of variation (CV) of 1.38. A CV greater than 1 indicates a high level of dispersion, suggesting significant variability in the efficiency values. This indicates that, in addition to the overall low efficiency, there is considerable variation in the efficiency across different enterprises.

However, the results from the first stage of DEA have not accounted for external environmental factors and random disturbances, and therefore, do not fully reflect the true operational management levels of the DMUs. To obtain a more accurate assessment of the true efficiency of the enterprises, further adjustments and recalculations are necessary to eliminate the effects of external interference and provide more reliable results.

### 4.4 SFA stage results: The influence of the environment on efficiency

#### 4.4.1 Design of environmental factors by PCA.

In this study, 23 internal and external environmental factors that could potentially affect a company’s operational efficiency but are beyond the company’s control were selected. PCA was applied to extract the principle components which were used as the independent variables (IV) for the SFA method.

(1) Data Suitability Assessment: To identify the environmental factors influencing the operational context, a total of 23 variables were collected and subjected to a suitability assessment for factor analysis. The Kaiser-Meyer-Olkin (KMO) measure yielded a value of 0.861, exceeding the acceptable threshold of 0.6, confirming the adequacy of the sample. Additionally, Bartlett’s test of sphericity (χ² = 70141, df = 253, p < 0.001) indicated significant correlations among the variables, further validating their suitability for factor analysis. The results, summarized in Table 6, support the feasibility of conducting the analysis, with a significance level of p < 0.05. Table 7 presents the KMO and Bartlett’s test results.

**Table 7:** KMO and Bartlett’s test.

Measure	Value
KMO Measure of Sampling Adequacy	0.861
Bartlett’s Test of Sphericity Approximate Chi-Squar	70141
Degrees of Freedom	253
Significance	0.000

(2) Principal Component Extraction: PCA was performed using SPSS, resulting in the extraction of 5 principal components with eigenvalues greater than 1. These components collectively accounted for 81.92% of the total variance, indicating a high level of explanation for the dataset. The total variance explained by the components is presented in Table 8.

**Table 8:** Eigenvalues and variance explained before and after rotation.

Component	Initial Eigenvalues			Extraction Sums of Squared Loadings			Rotation Sums of Squared Loadings		
	Total	% Variance	Cum. %	Total	% of Variance	Cum. %	Total	% of Variance	Cum. %
1	11.44	49.73	49.73	11.44	49.73	49.73	10.07	43.78	43.78
2	3.24	14.07	63.79	3.24	14.07	63.79	3.67	15.96	59.74
3	1.80	7.81	71.61	1.80	7.81	71.61	2.11	9.16	68.90
4	1.26	5.49	77.09	1.26	5.49	77.09	1.69	7.36	76.26
5	1.11	4.83	81.93	1.11	4.83	81.93	1.30	5.67	81.93
6	0.94	4.09	86.02	–	–	–	–	–	–
...	–	–	–	–	–	–	–	–	–
23	0.01	0.0032	100.00	–	–	–	–	–	–

After performing PCA, the eigenvalues and variance explained by the factors were presented in Table 7. However, due to the minimal differences in the variance percentages among some of the factors, it is challenging to clearly identify the relationships between the factors and the variables. To address this, further factor rotation was performed to increase the disparity in factor loadings, thereby enhancing the clarity of the factor structure. The results of the rotated component matrix are also presented in Table 9.

**Table 9:** Rotated component matrix.

Original Variable	Factor1	Factor2	Factor3	Factor4	Factor5
Total R&D Full-Time Hours	0.96	–	–	–	–
Annual R&D Investment	0.95	–	–	–	–
Government Support for Education	0.95	–	–	–	–
Annual New Product Sales	0.94	–	–	–	–
Local General Budget Revenue	0.93	–	–	–	–
Government Expenditure S&T	0.90	–	–	–	–
Authorized Utility Patent	0.88	–	–	–	–
Authorized Design Patent	0.87	–	–	–	–
Annual Number of R&D Projects	0.86	–	–	–	–
Government Expenditure on Pollution Treatment	0.80	–	–	–	–
Authorized Invented Patents	0.80	–	–	–	–
Disposable Income Per Capita	–	0.90	–	–	–
Consumption Level Per Capita	–	0.88	–	–	–
GDP Per Capita	–	0.87	–	–	–
*Air Pollution Emissions	–	0.67	–	–	–
*Water Pollution Emissions	−0.58	0.60	–	–	–
Population with High School Education	–	–	0.96	–	–
Population with Higher Education	–	–	0.96	–	–
Foreign Capital Registered	–	–	–	0.90	–
Foreign Direct Investment (FDI)	0.57	–	–	0.78	–
State-Owned Enterprise (SOE) Share	–	–	–	–	0.71
Firm Size	–	–	–	–	0.63
Ownership Concentration	–	–	–	–	0.58

Note: (1) Indicators marked with “*” have been adjusted to reflect their negative nature during data preprocessing; (2) In the table, “-” indicates that the rotated component did not reach the threshold of 0.5.

Based on the results of the rotated component matrix presented in Table 7, five common factors were identified, with variables showing loadings greater than 0.5 retained for factor interpretation.

**Z**_**1**_ **(Innovation and Innovation Environment):** This factor captures variables related to innovation inputs and outputs, as well as economic and policy support for innovation.

**Z**_**2**_ **(Living Standards and Environment):** This factor is associated with residents’ wealth and the quality of the living environment.

**Z**_**3**_ **(Labor Source for Pharmaceutical Industry)**: This factor reflects the levels of secondary and higher education, which are critical for providing talent suitable for the pharmaceutical companies.

**Z**_**4**_ **(Foreign Investment)**: This factor represents the extent of foreign investment in the region.

**Z**_**5**_ **(Internal Corporate Environment):** This factor encompasses variables related to corporate equity, company size, and ownership structure.

The five extracted environmental factors, Z_1_ to Z_5_, are employed as IV in the SFA analysis to reveal their influence on efficiency.

#### 4.4.2 The SFA results.

In this step, the five environmental factors were used as independent variables (IV), and the input slacks from the first stage as dependent variables (DV), to perform an SFA regression analysis. Table 10 presents the results of the SFA analysis in this step.

**Table 10:** Results of SFA regression of environmental factors on input slacks.

Independent Variable		Dependent Variable			
		Slack of Total Asset	Slack of Operation Cost	Slack of Employees Number	Slack of R&D Investment
Constant Term		−2161.54	−450.06	−864.36	−150.39
Z1: Innovation and Innovation Environment	β1	−1.68	−0.66^**^	−0.41	0.40^***^
	t-ratio	−1.17	−2.26	−1.01	4.17
Z2: Living Standards and Living Environment	β2	3.37	−2.58^***^	−5.64^***^	2.65^***^
	t-ratio	0.78	−2.63	−4.98	8.23
Z3: Labor Suitable for Pharmaceutical Industry	β3	−6.99^***^	−3.11^***^	−4.25^***^	1.38^**^
	t-ratio	−3.93	−3.11	−4.23	2.52
Z4: Foreign Investment Level	β4	15.47^***^	−3.73^***^	−8.93^***^	0.53
	t-ratio	7.52	−3.73	−8.44	0.53
Z5: Uncontrollable Internal Factors	β5	58.02^***^	15.43^***^	27.42^***^	−1.27^*^
	t-ratio	7.78	15.05	17.98	−1.88
σ²		17361819	1068720	8255823	72510
γ		0.999	0.999	0.999	0.999
LR test of the one-sided error		868	795	861	1048

Note: ^*^, ^**^, ^***^ indicate significance at the 10%, 5%, and 1% levels, respectively. Only coefficients with a significance level of 5% or lower (p ≤ 0.05) are included in subsequent calculations, while those above 5% (p > 0.05) are excluded.

As presented in Table 10 the likelihood ratio (LR) test values for the four input slack variables surpass the critical threshold of the mixed chi-square distribution and demonstrate significance at the 1% level, affirming the robustness of the model.

The γ values, which approach unity, suggest that input slacks are predominantly attributed to managerial inefficiency rather than stochastic noise. This finding underscores the potential for enterprises to enhance operational efficiency through managerial assessment and the strategic optimization of input slacks.

As shown in Table 9, both external environmental factors and internal uncontrollable factors have complex impacts on operational efficiency. For instance, innovation is negatively correlated with operational costs but positively associated with R&D investment. Improved living standards reduce costs and labor input while significantly enhancing R&D investment. Labor supply effectively decreases asset allocation, operational costs, and labor input, while promoting R&D investment. Foreign investment increases asset allocation but generally reduces costs and labor input. In contrast, internal uncontrollable factors, such as the proportion of state ownership and higher equity concentration, increase asset allocation, operational costs, and labor input while simultaneously reducing R&D investment. A detailed analysis of these effects is provided in Chapter 5.

To ensure that all DMUs can be evaluated more accurately under consistent environmental conditions, it is essential to exclude the effects of environmental factors and random disturbances in the analysis. The adjustment process is as follows: managerial inefficiency is calculated using Equation (4), followed by the estimation of random disturbances through Equation (5). Finally, the adjusted input values for each DMU are derived using Equation (6). Due to the complexity of this process, the detailed calculation steps are provided in the data availability section.

### 4.5 The result of Stage3: The obtain of the accurate efficiency

In Stage 3, the Undesirable Output DEA model was employed, with the adjusted inputs and initial outputs substituted into the CRS and VRS models. All results are presented in Table 5.

After excluding the environmental influences, the TE increased from 0.202 to 0.227. Despite this improvement, the TE remains relatively low. The number of effective DMUs increased from 144 to 167, accounting for 9.57% of all the samples.

Regarding the distribution of efficiency, even after accounting for environmental factors, the SD is 0.279, and the coefficient of variation remains at 1.31, indicating a significant disparity in efficiency between firms and reflecting imbalances in management levels.

At the regional level, after removing the environmental factors, the Central China region exhibited the highest technical efficiency at 0.286, while the differences in efficiency across the Northeast, South China, Southwest, and East China regions were relatively small. The Northwest and North China regions had the lowest efficiency values, with the pharmaceutical industry in the Northwest being relatively underdeveloped and the sample size smaller, which may introduce some bias. Additionally, the Central China region had the highest pure technical efficiency at 0.316, while the Northwest region had the lowest pure technical efficiency, with minimal differences across other regions.

In terms of changes in technical efficiency, both the Northwest and North China regions experienced a decline, suggesting that the efficiency in the first stage may have been overestimated, while other regions showed some degree of underestimation. This indicates that, given the current environment, the regions of North China and Northwest are more conducive to improving pharmaceutical efficiency, while other environmental factors are somewhat inhibiting the overall efficiency of pharmaceutical enterprises. The factors driving these changes will be discussed in detail in Chapter 5.

Regarding changes in pure technical efficiency, a decrease was observed across all regions after eliminating environmental factors. This indicates that although external environmental influences have been accounted for, the improvement in firms’ pure technical capabilities or internal management remains limited. Thus, it is essential to investigate and address bottlenecks in technological innovation, resource allocation, and management processes to achieve sustained improvements in efficiency.

### 4.6 Impact of internal resource allocation on efficiency based on the tobit model

After performing dimensionality reduction with PCA to extract external and uncontrollable internal factors, and subsequently applying SFA regression, the impact of these factors on efficiency was revealed. Meanwhile, after excluding these factors, a decrease in the nationwide average PTE was observed, indicating inefficiencies in the industry’s internal resource allocation. Therefore, it is crucial to analyze internal resource allocation and to reveal its effect on efficiency. Therefore, In this stage, the final efficiency values are used as the dependent variable, while the internal resource allocation methods adopted by enterprises serve as the independent variables for Tobit regression analysis.

#### 4.6.1 Independent variables applied in this study.

This study adopts the following resource allocation methods as independent variables for the Tobit regression, based on the input variables of the DEA model.

Firstly, for total assets, this study considers the proportion of fixed assets and the asset-liability ratio as independent variables. The allocation of fixed assets directly determines asset density, while the asset-liability ratio serves as an indicator of the firm’s financial leverage, influencing the risk-return tradeoff in asset utilization. Consequently, the asset-liability ratio is a crucial metric for assessing a firm’s financial structure and risk management capabilities.

Secondly, with respect to operational costs, while certain expenditures are unavoidable, the allocation of these costs can significantly impact the overall profitability of the firm. This study employs the ratio of management expenses, sales expenses, and R&D expenses to operating income as independent variables to evaluate the firm’s resource allocation efficiency and cost management practices across different domains.

Finally, regarding personnel allocation, this study examines both the distribution of positions and the educational background of employees. The distribution of positions reflects the efficiency of resource allocation across various roles within the firm, while the educational level of employees influences their productivity and innovative capacity.

#### 4.6.2 Tobit regression results.

Given the efficiency obtained from DEA is confined to the (0, 1], the use of ordinary least squares (OLS) estimation may result in significant bias. To address this, the Tobit regression model, a widely applied econometric approach designed for analyzing truncated data, is employed in this study. Accordingly, a regression model is developed to conduct empirical analysis.

This study conducted regression analysis using Stata 17.0 software. Prior to the regression, all independent variables were tested for multicollinearity, and all VIF values were below 5, indicating that multicollinearity was not a significant concern in the model. Table 11 illustrates the impact of the eight resource allocation methods on technical efficiency.

**Table 11:** Regression results of resource allocation methods influencing TE.

Independent Variables	Coefficient	Standard Error	T-value	P-value
Constant	0.269^***^	0.056	4.90	0.000
Fixed Asset Ratio	−0.111	0.104	−1.08	0.282
Debt Ratio	0.071	0.071	1.01	0.312
Management Expense Ratio	−0.554^***^	0.180	−3.08	0.002
Sales Expense Ratio	0.230^***^	0.081	2.82	0.005
Production Staff Ratio	−0.065^***^	0.026	−2.61	0.009
Technical Staff Ratio	−0.252^**^	0.129	−1.96	0.050
Sales Staff Ratio	−0.106	0.082	−1.29	0.197
Higher Education Ratio	0.213^**^	0.085	2.51	0.012

Note: ^***^ and ^**^ represent 99% and 95% confidence levels, respectively,

The likelihood ratio test indicates that the Tobit model is statistically significant overall (p < 0.05), suggesting that the independent variables collectively have a significant impact on the dependent variable. Based on the regression results in Table 10, resource allocation methods overall have a significant impact on efficiency. Specifically, the management expense ratio, sales expense ratio, production staff ratio, and technical staff ratio have significant effects on technical efficiency (TE) at the 99% confidence level, with coefficients of −0.554, 0.230, −0.065, and −0.252, respectively. Additionally, the higher education ratio shows a significant effect at the 95% confidence level, with a coefficient of 0.213. However, the fixed asset ratio, debt ratio, and sales staff ratio are not statistically significant, as their p-values exceed 0.05.

From the perspective of asset allocation, the fixed asset ratio and debt ratio do not have significant impacts on technical efficiency (p > 0.05). This suggests that neither the physical allocation of assets nor the use of financial leverage has a notable effect on efficiency improvement.

From the perspective of expense allocation, an increase in management expenses significantly reduces technical efficiency (coefficient = −0.554, p < 0.01), with a relatively large absolute coefficient, indicating a more pronounced negative impact. In contrast, an increase in sales expenses significantly enhances technical efficiency (coefficient = 0.230, p < 0.01), suggesting that investments in sales-related activities can drive efficiency improvements, potentially due to better market promotion or optimized sales strategies.

From the perspective of staff allocation, simply increasing the proportion of production and technical staff reduces technical efficiency (coefficients = −0.065 and −0.252, respectively, both significant at p < 0.01). This may be because the pharmaceutical industry is highly innovation-driven, and relying solely on increasing staff numbers does not resolve efficiency issues. Instead, it may lead to inefficiencies due to suboptimal resource allocation. Improving the educational level of employees, however, significantly enhances technical efficiency (coefficient = 0.213, p < 0.05), indicating that raising the overall quality and skill level of employees enables firms to achieve innovation-driven and efficiency-optimized outcomes more effectively.

### 4.6.3 Robustness test of methods

The results of the previous three-stage DEA analysis show that pure technical efficiency generally decreased after accounting for environmental factors. To further verify the robustness of the model, this study uses PTE as the dependent variable while maintaining consistency in the explanatory variables for regression analysis. And the robustness verification result is listed as in Table 12.

**Table 12:** The robustness verification result.

Independent Variables	Coefficient	Standard Error	T-value	P-value
Constant	0.277^***^	0.067	4.11	0.000
Fixed Asset Ratio	−0.206	0.135	−1.52	0.128
Debt Ratio	0.098	0.089	1.10	0.270
Management Expense Ratio	−0.683^***^	0.217	−3.15	0.002
Sales Expense Ratio	0.281^***^	0.099	2.83	0.005
Production Staff Ratio	−0.021	0.035	−0.6	0.546
Technical Staff Ratio	−0.270	0.167	−1.62	0.106
Sales Staff Ratio	−0.071	0.105	−0.67	0.501
Higher Education Ratio	0.255^**^	0.116	2.20	0.028

The Tobit regression model demonstrates robust results. The overall model is significant (Prob > F = 0.00), with no severe multicollinearity (VIF < 5). Key variables such as management expense ratio, sales expense ratio, and higher education ratio are significant, with coefficients aligning with theoretical expectations. The use of robust standard errors addresses potential heteroscedasticity, and the residual variance is small, indicating stable estimates. While some variables are not significant, they do not compromise the model’s overall robustness.

In summary, the allocation of internal resources significantly impacts efficiency. For TE, increasing the proportion of sales expenses and improving personnel quality (e.g., the proportion of highly educated employees) enhances efficiency, while higher management expenses and certain personnel proportions (e.g., technical and production staff) may reduce it. This provides a theoretical foundation for optimizing resource allocation within enterprises and empirical support for resource distribution decisions.

## 5 Discussion

Based on the results, this chapter discusses five key aspects: the temporal changes in overall efficiency and their underlying causes, the distribution characteristics of overall efficiency, the efficiency changes between the first and third stages along with their potential reasons, the influence of external and internal uncontrollable factors on efficiency, and the role of internal resource allocation in shaping efficiency.

### 5.1 Temporal changes and causal analysis

**Fig 2:**
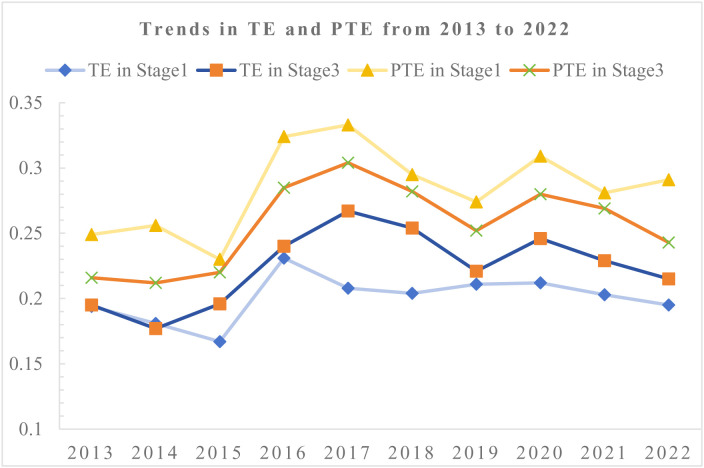
Trends in TE and PTE between stage 1 and stage 3 from 2013 to 2022.

By calculating the average efficiency of each DMU over a ten-year period, the overall performance was evaluated, and intertemporal variations in efficiency were systematically compared. The aggregated results of TE and PTE for different years are illustrated in Fig 2, highlighting the temporal dynamics of efficiency changes and offering a foundation for underlying the causes.

The data from the chart reveals that the overall efficiency of Chinese pharmaceutical enterprises, analyzed as a cross-sectional dataset, has not demonstrated significant improvement over the past decade. Both TE and PTE peaked in 2017, followed by a gradual decline. This trend aligns with the onset of major pharmaceutical system reforms in 2017, such as the introduction of the Implementation Plan for Consistency Evaluation of Generic Drugs, which mandated quality and efficacy evaluations for generics. Subsequently, pilot programs for centralized procurement of generic drugs were launched in 2018 and expanded further in 2019. While these reforms aimed to standardize industry practices, they constrained efficiency improvements, particularly for pharmaceutical enterprises with inherently weak competitiveness. The increased regulatory pressures further exacerbated efficiency declines in these firms.

In 2020, the outbreak of the COVID-19 pandemic temporarily improved operational performance for certain enterprises under strong government intervention, resulting in a short-lived rise in efficiency. However, this improvement was not sustained, as efficiency declined once the pandemic’s initial impact subsided.

The most significant gap between efficiency levels in Stage 1 and Stage 3 was observed during 2017–2018, coinciding with the period of intensive policy reforms in the pharmaceutical sector. This suggests that, beyond the regulatory influence of pharmaceutical authorities, broader environmental factors such as intensified market competition and escalating cost pressures also negatively affected enterprise efficiency. The combined effects of policy-driven pressures and external uncertainties have presented significant challenges to efficiency improvements within the industry.

### 5.2 Efficiency distribution of chinese pharmaceutical enterprises across provinces

**Fig 3:**
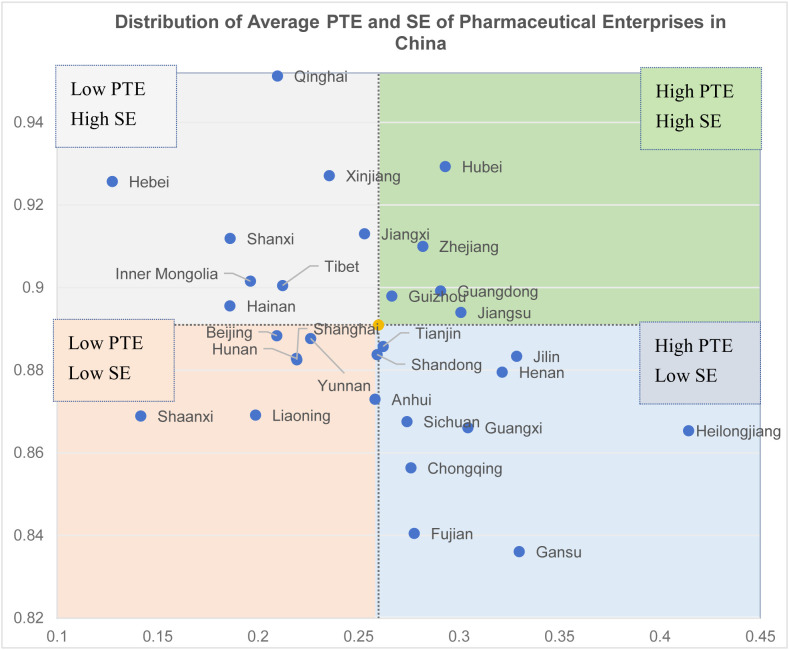
Average PTE and SE distribution of pharmaceutical enterprises across Chinese provinces.

In the results section, this study highlights that, in both the first and third stages, efficiency differences among enterprises are statistically significant, with coefficients of variation exceeding 1.3. This indicates a high degree of dispersion in the efficiency distribution across enterprises. To further investigate interprovincial differences in enterprise efficiency and the underlying causes of uneven development, this study uses the overall average values of PTE and SE as thresholds. Based on these thresholds, the average efficiency of enterprises in each province is categorized into four distinct types, as illustrated in Fig 3. This classification evaluates provinces according to their relative positions in PTE and SE performance, providing a more detailed perspective for analyzing regional disparities.

The pharmaceutical enterprises in the first-tier regions demonstrate high PTE and SE, including Zhejiang, Guangdong, Jiangsu, Hubei, and Guizhou. These regions exhibit strong resource allocation capabilities and significant scale effects, resulting in high overall efficiency.

The underlying reasons for their efficiency vary: Zhejiang, Guangdong, and Jiangsu benefit from advanced economies, substantial R&D investments, and access to international markets, enabling enterprises to achieve large-scale production and technological innovation. Hubei leverages abundant academic and research resources, fostering technological advancements through industry-university-research collaboration. Guizhou capitalizes on lower labor costs and strong government support to optimize resource utilization. These regions have developed unique high-efficiency models through the synergistic effects of technology, resources, and policies.

The pharmaceutical enterprises in the second-tier regions demonstrate high PTE but relatively low SE. These regions include Heilongjiang, Jilin, Sichuan, and Chongqing and others. While these enterprises exhibit strong technical capabilities and efficient resource allocation, their underperformance in realizing scale economies affects their overall efficiency.

The underlying causes are multifaceted. First, the production scale of these enterprises does not fully align with market potential, leading to suboptimal scale efficiency. Additionally, the economic development levels and market demand in these regions are relatively fragmented, constraining enterprises’ ability to expand scale and optimize resource integration. For instance, Sichuan and Chongqing have a significant number of listed companies, but their scale efficiency lags behind that of Zhejiang and Jiangsu. Similarly, although Heilongjiang and Liaoning benefit from a strong talent pool, their pharmaceutical industries remain less developed compared to Zhejiang and Jiangsu, with weaker industrial integration and supply chain optimization. Furthermore, delayed regional policy support and infrastructure development exacerbate these challenges. To enhance overall efficiency, these enterprises need to optimize resource allocation, strengthen supply chain management, and foster innovation to improve scale economies.

The pharmaceutical enterprises in the third-tier regions exhibit High SE but relatively low PTE. These regions include Hebei, Tibet, Qinghai, Shanxi, among others. While these enterprises benefit from large production scales and significant scale economies, their limitations in technology utilization and resource optimization constrain overall efficiency.

Possible reasons include reliance on traditional production methods, leading to weak innovation capacity and lower resource utilization efficiency. Insufficient R&D investment and a shortage of high-skilled talent further hinder improvements in PTE. Additionally, geographic remoteness or weaker economic foundations in some areas make it challenging to attract advanced technologies and external resources. For example, certain enterprises in Tibet demonstrate notable SE but lag in technological adoption and management practices. To address these issues, enterprises should increase R&D investment, optimize production management, and leverage advanced technologies and external resources to enhance PTE and overall competitiveness.

The pharmaceutical enterprises in the fourth-tier regions, including Beijing, Shanghai, Yunnan, Shaanxi, Hunan, and Liaoning, exhibit low technical efficiency (TE) and pure technical efficiency (PTE). These enterprises show overall low efficiency, with significant potential for improvement in technology utilization and resource allocation.

In Beijing and Shanghai, possible reasons for low efficiency include two main factors. First, relatively strict environmental and safety regulations may impose higher compliance costs, potentially slowing down technological upgrades and innovation. Second, the high cost of human resources in these economically advanced regions could add financial pressure on enterprises, limiting their ability to allocate resources effectively. Meanwhile, in regions such as Yunnan, Shaanxi, Hunan, and Liaoning, weaker economic foundations, insufficient R&D investment, and a shortage of high-skilled talent remain primary challenges. Geographic remoteness and structural barriers to technology diffusion further exacerbate these inefficiencies. Addressing these issues requires tailored strategies to improve resource allocation and foster innovation.

### 5.3 Changes in efficiency during stage 1and stage 3

**Fig 4:**
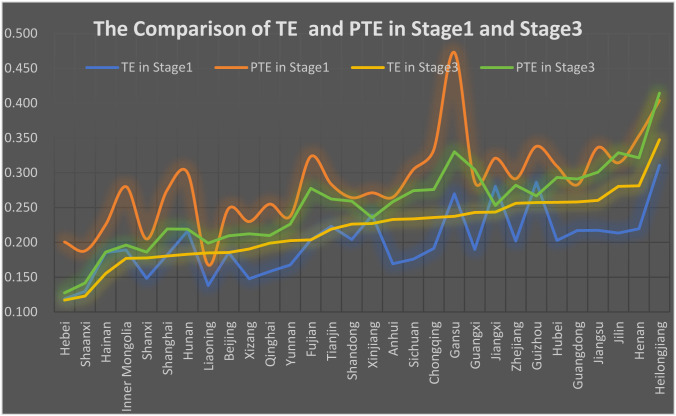
The comparison of TE and PTE in stage 1 and stage 3.

After the exclusion of environmental factors in the second stage by SFA, it was observed that the average TE of pharmaceutical companies in 11 provinces declined, indicating that their efficiency in the first stage was overestimated under current environmental conditions. Among these, Jiangxi, Hunan, Gansu, Hainan, and Guizhou experienced similar levels of efficiency reduction. This suggests that favorable environmental factors in the first stage, such as low labor costs, lenient policy environments, or minimal environmental regulations, contributed to the overestimation of efficiency. Additionally, it highlights that external environmental conditions and some uncontrollable internal factors currently provide significant operational support in these regions. Notably, these provinces are generally economically underdeveloped, with an immature pharmaceutical industry. However, they possess development potential due to abundant labor resources, policy support, well-established infrastructure, and relatively lenient regulatory environments. Fig 4 presents the changes in TE and PTE in Stage 1 and Stage 3.

On the other hand, the average TE in 17 provinces increased, indicating that their efficiency was underestimated in the first stage. Among these, Jilin, Anhui, Henan, and Sichuan showed the most significant improvement. This reflects that pharmaceutical enterprises in these regions face certain unfavorable environmental conditions under current circumstances. Specifically, while the pharmaceutical industry in these provinces has a certain foundation, they lag in innovation capacity, living standards, and openness to external markets. Overall, these regions are characterized by large populations and complex market demands, with relatively stringent regulatory frameworks that impose additional constraints on operational efficiency. After adjusting for environmental factors, the results more accurately reflect the intrinsic technical and managerial efficiency of these enterprises. This suggests that, despite external environmental limitations, these regions have significant potential for improving technical efficiency and operational performance. Efforts to enhance innovation capacity, optimize regulatory frameworks, and increase openness to external markets can further improve their overall efficiency.

### 5.4 The impact of environment on efficiency

#### (1) The impact of innovation and innovation environment on efficiency.

Regional innovation significantly reduces operational costs (t = −2.26) while increasing R&D investment (t = 4.17). This suggests that innovation optimizes production processes and enhances resource efficiency by minimizing inefficiencies. However, advancing innovation requires substantial investment, such as technology development, equipment upgrades, and talent acquisition, which increases R&D expenditures. This reflects a balance between short-term cost savings and long-term technological advancements.

#### (2) The impact of living standards and environment on efficiency.

Higher living standards significantly reduce operational costs (t = −2.63) and employee numbers (t = −4.98), while greatly increasing R&D investment (t = 8.23). This indicates a shift from labor-intensive to technology-intensive production models as firms adapt to higher labor costs in regions with better living conditions. The demand for advanced technologies and capital-intensive strategies in these regions enhances productivity and drives innovation.

#### (3) The impact of labor suitable for the pharmaceutical industry on efficiency.

Labor supply significantly reduces asset allocation (t = −3.93) and operational costs (t = −3.11), while positively influencing R&D investment (t = 2.52). This highlights the role of an adequate and skilled labor force in meeting production needs while supporting innovation. The efficiency gains from labor availability are particularly evident in industries requiring highly skilled workers, such as pharmaceuticals, where labor quality directly impacts innovation outcomes.

#### (4) The impact of foreign investment level on efficiency.

Foreign investment increases asset allocation (t = 7.52) but significantly reduces operational costs (t = −3.73) and employee numbers (t = −8.44). This reflects a preference for advanced technologies and efficient management practices that optimize production and reduce reliance on low-skilled labor. Foreign firms’ capital-intensive strategies, driven by high return expectations, contribute to technological upgrades and efficiency improvements, fostering long-term competitiveness.

#### (5) The impact of uncontrollable internal factors on efficiency.

Internal factors, such as a high proportion of state ownership, equity concentration, and company size, increase asset allocation (t = 7.78), operational costs (t = 15.05), and labor input (t = 17.98) while suppressing R&D investment (t = −1.88). This suggests inefficiencies in resource utilization due to rigid governance structures and risk-averse decision-making. The focus on short-term goals over long-term innovation in such structures constrains technological progress and reduces competitiveness in dynamic markets.

In Summary, External factors, such as innovation, living standards, labor supply, and foreign investment, generally contribute to optimizing resource allocation and improving efficiency, albeit with varying degrees of impact. In contrast, internal uncontrollable factors often result in resource misallocation and inefficiencies, underscoring the importance of institutional reform and governance optimization to enhance operational efficiency and innovation capacity.

### 5.5 The impact of internal resource allocation methods on efficiency

#### (1) The impact of asset allocation on efficiency.

Both the Fixed Asset Ratio and Debt Ratio are not statistically significant, indicating that asset allocation methods have a limited impact on efficiency.

#### (2) The impact of expense allocation on efficiency.

The Management Expense Ratio is significantly negatively correlated with efficiency (t = −3.08), likely because excessive management expenses increase operational burdens, diverting resources to low-efficiency activities. In contrast, the Sales Expense Ratio is significantly positively correlated with efficiency (t = 2.82), suggesting that sales investments effectively expand market share and optimize resource utilization, thereby improving overall efficiency.

#### (3) The impact of personnel allocation on efficiency.

The Production Staff Ratio is significantly negatively correlated with efficiency (t = −2.61), potentially due to excessive production staff, which leads to diminishing returns on labor resources. Similarly, the Technical Staff Ratio is significantly negatively correlated with efficiency (t = −1.96), possibly reflecting the high cost of technical personnel that may not translate into immediate efficiency gains. The Sales Staff Ratio shows no significant correlation with efficiency, indicating that its marginal benefit may be limited or the allocation of sales staff resources may not be fully effective. In contrast, the Higher Education Ratio is significantly positively correlated with efficiency (t = 2.51), demonstrating that highly educated employees optimize resource allocation, enhance decision-making, and foster innovation, thereby improving overall efficiency.

In summary, internal resource allocation methods exhibit varied impacts on efficiency. Asset allocation shows limited influence, while management expenses reduce efficiency. Conversely, sales expenses and a higher proportion of highly educated employees significantly enhance efficiency. Optimizing expense allocation and workforce composition is critical to improving operational efficiency.

## 6. Conclusion

### 6.1 Main finding

China’s listed pharmaceutical companies demonstrate relatively low overall efficiency, with significant regional disparities. Over the past decade, efficiency improvements have been limited, peaking in 2017 and subsequently declining. External environment, such as innovation, living standards, labor supply, and foreign investment, have played a crucial role in enhancing efficiency. However, internal factors, including state ownership, company size, and equity concentration, have had a negative effect on efficiency. In terms of internal resource allocation, asset allocation shows a limited impact on efficiency, while management expenses hinder performance. Conversely, increased sales expenses and a higher proportion of highly educated employees significantly enhance efficiency.

### 6.2 Relationship between findings and existing theories

This study finds that the overall operational efficiency of pharmaceutical enterprises in China is relatively low, with significant disparities in development both between enterprises and across regions. This conclusion aligns with current evaluations based on single-dimensional assessments [57,93].

The research highlights that external environmental factors play a critical role in influencing regional pharmaceutical efficiency. Porter’s theory of competitive advantage emphasizes that market demand, technological innovation, and competitive pressure are key drivers of industrial upgrading and efficiency enhancement. Furthermore, Porter’s regional economic theory underscores the significant synergies in industrial development across regions. The integration of industrial chains can foster overall regional economic growth and enhance competitiveness. This synergy manifests in the sharing of innovation resources, talent mobility, and capital allocation, thereby exerting both direct and indirect positive effects on firm efficiency.

Existing literature predominantly focuses on the multidimensional influence of external and internal factors—such as innovation capacity, income levels, labor supply, foreign direct investment (FDI)—on corporate efficiency [53,57,64]. However, most studies tend to analyze single variables, potentially overlooking the complex interrelationships among various environmental factors and their combined impact on firm efficiency.

This study employs PCA to conduct dimensionality reduction on multiple external environmental variables, extracting core components that explain major fluctuations in the external environment. This approach transcends the reliance on single-variable analyses in previous studies, providing a more comprehensive and systematic reflection of the overall impact of the external environment on firm efficiency. Consequently, the method mitigates potential biases or omissions associated with single-variable analyses. Through this methodological innovation, the study enriches the existing theoretical framework, offering new analytical perspectives and empirical evidence for understanding how complex external environments influence corporate efficiency through multiple pathways.

In revealing the impact of resource allocation on efficiency, how to optimize resources to increase the efficiency is a central focus of this study. based on the Resource-Based View (RBV), the core competitiveness of the companies is from rare and inimitable internal resources and capabilities, including tangible assets, intangible assets, and human capital [21]. Proper allocation of these resources is crucial for improving firm performance [24,29].

Research on the pharmaceutical industry indicates that asset allocation methods have no significant effect on operational efficiency. However, the distribution of management and sales expenses, as well as human resource allocation, significantly influences firm efficiency. In particular, increasing sales investment and raising the proportion of highly educated employees positively impact operational efficiency, aligning with the core tenets of the Resource-Based View and human capital theory.

### 6.3 Implication of this study

This study reveals that the overall efficiency of China’s pharmaceutical enterprises is relatively low, with significant regional disparities. Both external environmental factors and internal resource allocation have a notable impact on efficiency. Accordingly, the following implication from this study are proposed for policymakers and management:

#### (1) Implications for policymakers.

Firstly, the local innovation environment exhibits a suppressive effect on asset and labor input, while failing to significantly promote R&D investment. This suggests that government efforts to drive industrial innovation should focus on optimizing the innovation ecosystem, rather than relying solely on factor input expansion. Policy measures such as tax incentives, R&D subsidies, and fostering collaboration between enterprises and research institutions should be strengthened to enhance firms’ intrinsic innovation capacity.

Secondly, high living costs and stringent environmental regulations pose operational challenges for pharmaceutical companies. However, this does not imply that standards for quality of life or environmental protection should be compromised. Instead, the government should play a guiding role in spatially restructuring the pharmaceutical industry. Labor-intensive and pollution-prone segments of the value chain should be relocated to regions with lower operational costs and greater environmental carrying capacity.

Thirdly, the availability of labor resources has a positive impact on firm efficiency. The government should therefore invest in the cultivation and technical training of pharmaceutical professionals. This can be achieved by enhancing industry-academia collaboration and building an integrated talent development system to support enterprise-level innovation and long-term growth.

Fourthly, increasing the level of openness to trade and investment improves the efficiency of asset allocation and cost control. Pharmaceutical firms are encouraged to engage in international collaboration and expand their global presence. For central and western regions with relatively low openness, governments should improve the business environment, simplify administrative procedures, and offer tax incentives to attract foreign investment into the pharmaceutical sector.

Fifthly, internal firm characteristics—such as state ownership, equity concentration, and enterprise scale—negatively affect operational efficiency. Policymakers are advised to advance governance reforms in state-owned enterprises (SOEs), reduce inefficient state holdings, and encourage the adoption of market-oriented management mechanisms to improve overall firm performance.

In conclusion, the empirical findings of this study provide theoretical support for policy formulation and industry governance. Government interventions should focus on optimizing the innovation environment, enhancing the talent pipeline, rationalizing industrial spatial distribution, attracting foreign capital, and promoting SOE reform to comprehensively improve the operational efficiency and global competitiveness of China’s pharmaceutical industry.

#### (2) Implications for management.

Optimizing Expenses Structures: The company should reduce unnecessary management expenses, such as administrative costs, and streamline redundant personnel, ensuring that funds are directed towards departments that drive business growth, such as sales. At the same time, in terms of sales, efforts should be made to strengthen market expansion, improve product quality, and enhance brand influence. By combining internal cost reduction with increased market investment, overall operational efficiency can be improved.

Optimizing Workforce Structure: The company should prioritize talent quality over quantity. By improving employees’ education levels and professional skills, and recruiting highly educated and skilled personnel, the company can enhance its efficiency.

Increasing Asset Utilization: Companies should make rational use of assets to avoid over-investment and idleness. By optimizing production processes and improving equipment utilization, unnecessary fixed asset investments can be reduced. At the same time, for idle assets, companies can actively expand their business, such as through CRO/CDMO, to achieve business diversification and enhance corporate competitiveness.

In summary, governments and corporate management should work in collaboration to address both external environmental factors and internal operational efficiencies, thereby enhancing the overall productivity of the pharmaceutical sector and ensuring sustainable industry development.

### 6.4 Research limitations and future research

#### (1) Research limitations.

This study focuses solely on publicly listed pharmaceutical companies in China, excluding unlisted companies and international pharmaceutical firms, which limits the generalizability of the findings. Additionally, the DEA model used in this research is sensitive to outliers and lacks statistical significance testing mechanisms, potentially affecting the reliability of the results. The study adopts a static analytical approach and does not consider the long-term impacts of technological progress or external environmental changes on efficiency. Moreover, the study does not analyze efficiency differences across sub-industries within the pharmaceutical sector, such as chemical pharmaceuticals, biopharmaceuticals, CDMO, and traditional Chinese medicine, which limits the applicability of the findings and policy recommendations. Lastly, from a methodological perspective, the three-stage DEA employed in this study is a static analytical approach and does not capture the dynamic changes in efficiency over time.

#### (2) Future research.

Future research should expand the sample scope to include unlisted companies and international pharmaceutical firms to enhance the generalizability of the findings. Additionally, methods such as bootstrap techniques can be used to improve the robustness of DEA efficiency scores. Incorporating dynamic analytical frameworks, such as the Malmquist Productivity Index, would allow for a more comprehensive understanding of efficiency changes over time, accounting for the effects of technological progress and environmental factors. Further classification of the pharmaceutical industry by core business areas, such as chemical pharmaceuticals, biopharmaceuticals, CDMO, and traditional Chinese medicine, will allow for more detailed and specific analyses, leading to more targeted policy recommendations for each sector. And lastly, the future research could consider incorporating the dynamic Malmquist productivity index to more comprehensively assess changes in enterprise efficiency over time and technological progress, thereby enhancing the timeliness and explanatory power of the efficiency evaluation.
